# Supplementary material for: Lycopene in Combination with Insulin Triggers Antioxidant Defenses and Increases the Expression of Components That Detoxify Advanced Glycation Products in Kidneys of Diabetic Rats
Source: Nutrients. 2024 May 23;16(11):1580. doi: 10.3390/nu16111580 (PMC11173891; doi:10.3390/nu16111580)
Supplement: Supplementary file 1 [file nutrients-16-01580-s001.zip › nutrients-2998799-supplementary.pdf]

Supplementary Table S1: Purchase, dilution and RRID of primary and secondary antibodies.

Primary antibodies	Company	Catalog No.	Batch	Dilution	RRID*
AGER-1 (DDOST)	Elabscience®	E-AB-63923	-	1:750	AB_2818987
GLO 1	Elabscience®	E-AB-15072	-	1:500	AB_2818989
Anti-β-actin	Cell Signaling	#5057	15	1:1000	AB_10694076
Anti-AKT (pan)	Cell Signaling	#4691	20	1:1000	AB_915783
Anti-Phospho-[Ser 473]-AKT	Cell Signaling	#4060	16	1:1000	AB_2315049
**Secondary antibodies**	**Company**	**Catalog No.**	**Batch**	**Dilution**	**RRID***
Anti-rabbit IgG, HRP-linked antibody	Cell Signaling	#7074	28	1:1000	AB_2099233
Anti-mouse IgG, HRP-linked antibody	Sigma-Aldrich	A5441	-	1:5000	-

* RRID: Research Resource Identifier
